# Trapping Asian Citrus Psyllid (*Diaphorina citri*) on Adhesive-Coated New Shoots of *Murraya paniculata*

**DOI:** 10.3390/insects16101011

**Published:** 2025-09-29

**Authors:** Ruimin Zhang, Yongjing Huang, Guiming Deng, Congyi Zhu, Pingzhi Wu, Zhengyan Fan, Jiwu Zeng

**Affiliations:** Institute of Fruit Tree Research, Guangdong Academy of Agricultural Sciences, Key Laboratory of South Subtropical Fruit Biology and Genetic Resource Utilization, Ministry of Agriculture and Rural Affairs, Guangdong Provincial Key Laboratory of Science and Technology Research on Fruit Tree, Guangzhou 510640, China; xmingz@163.com (R.Z.); fanzhengyan@gdaas.cn (Z.F.)

**Keywords:** Asian citrus psyllid, *Murraya paniculata*, adhesive, trap cropping, population dynamics, directional preferences

## Abstract

**Simple Summary:**

Asian citrus psyllids are the main vectors of citrus Huanglongbing (citrus greening disease). New shoots of orange jasmine (*Murraya paniculata*) sprayed with adhesive can more effectively trap Asian citrus psyllid (*Diaphorina citri*) adults, particularly females, than traditional yellow sticky traps. We report a male-biased Asian citrus psyllid sex ratio for a near-natural population and show that spraying adhesive on new shoots of orange jasmine can be used to determine the population dynamics and directional preferences of Asian citrus psyllids.

**Abstract:**

The Asian citrus psyllid (ACP), *Diaphorina citri* Kuwayama (Hemiptera: Liviidae), is a vector of *Candidatus* Liberibacter asiaticus (*C*Las), the causal agent of citrus Huanglongbing (HLB). We examine the effectiveness of spraying adhesive on new shoots of orange jasmine (*Murraya paniculata*) to trap ACP in laboratory and field conditions and for the monitoring of ACP population dynamics and directional preferences. After 36 h of observation, orange jasmine plants with new shoots, with and without adhesive, are significantly (*p* < 0.05) more attractive to ACP than plants without new shoots. In field trials, orange jasmine with new shoots attracted more ACP, particularly females, than plants without new shoots. A male-biased ACP sex ratio occurred in a near-natural population. Orange jasmine with new shoots coated with adhesive more effectively trapped ACP than yellow sticky traps, particularly during the winter and early spring, when ACP densities were low. ACP has a strong phototropic response, preferring to feed and rest in south- and east-facing positions. Adhesive trapping shows potential for attracting adult ACP, especially in citrus orchards during cooler seasons, when host trees lack new shoots, and it may be particularly effective in doing so in urban areas and unmanaged citrus refugia (the primary sources of ACP infestations for commercial groves).

## 1. Introduction

The Asian citrus psyllid (ACP), *Diaphorina citri* Kuwayama (Hemiptera: Liviidae), a major pest in citrus worldwide, is the primary vector of the bacterium *Candidatus* Liberibacter asiaticus (*C*Las), responsible for Huanglongbing (HLB) [1], also known as citrus greening disease. Because no effective control method is known, HLB is a globally devastating disease in citrus [2,3]. Controlling ACP is widely considered to be one of the most effective strategies to curb the spread of HLB [4]. Accordingly, the prevention, control, and dynamic monitoring of ACP is important for citrus industry development.

Chemical pesticides are commonly used in agriculture to control ACP, but grower reliance on these pesticides contributes to insecticide resistance [5,6,7]. Additionally, pesticide application causes problems such as excessive chemical residues in fruits, biodiversity loss, and environmental pollution [8]. More ecologically friendly ways to combat ACP are needed for the sustainable control of ACP populations.

Sexually mature female ACP individuals lay eggs on the tips of growing shoots or in the crevices of unfolded “feather flush” leaves, and they are strongly attracted to host plants with new shoots [4,9]. Citrus undergo several cycles of shoot growth (flushes) throughout the year, with most citrus trees having two to five such flushes from early spring to late summer [10,11]; intervals between flushes are longer in winter. After emergence, female ACP individualssexually mature within 3–5 d [12] and begin to search for suitable places to lay eggs after mating.

Habitat manipulation and diversification, including methods like trap cropping that strategically use other plants to attract and intercept target insect populations, can effectively and sustainably manage pests while minimizing damage to economically valuable crops [13,14,15]. Because orange jasmine (*Murraya paniculata*) is also a preferred host of ACP [16], and it can rapidly sprout with ample water and fertilizer following pruning, it represents an ideal host plant to use to attract ACP during intervals without citrus flushes [17]. Thus, using orange jasmine as a trap crop may effectively control ACP.

We investigate the efficacy of applying a sticky adhesive to new shoots of orange jasmine to attract ACP, as well as the sex ratio of the ACP population. We contrast this approach with yellow sticky traps to assess its practicality in monitoring ACP populations and directional preferences.

## 2. Materials and Methods

### 2.1. Materials

Experimental orange jasmine plants were cultivated in pots, including two-year-old plants (~30 cm height) and five-year-old plants of 100–120 cm (age 5 y). Plant specifications are detailed in Section 2.2.1 (2-year-old) and Section 2.3, Section 2.4, Section 2.5 (5-year-old). Plants in field experiments occurred along roadsides and measured ~120 cm height. The sticky spray adhesive (Super 75 Repositionable Adhesive, the main component of which is synthetic rubber, with solid content of 13%, volatile organic compounds (VOCs) < 55%, and a coating rate of 7.2 m^2^ L^−1^) was produced by 3M China Co., Ltd. (Shanghai, China).

The adhesive was sprayed 3 h before the experiment. During application, one hand stabilized the bottle base while the other pressed the actuator straight down, keeping the nozzle ~20 cm from the surface and moving it in uniform sweeping motions. The spraying interval was maintained between 24 and 30 h, depending on the adhesive viscosity.

### 2.2. Trapping Efficiency Evaluation

#### 2.2.1. Laboratory Experimentation Evaluation

The tops of orange jasmine plants of ~30 cm were pruned to initiate new shoot growth. When new shoots had grown ~5–10 mm, the adhesive was sprayed evenly on the new shoots or directly on old leaves. Spraying was repeated 2 or 3 times until the leaves were sticky to the touch. Four treatments were established: top-pruned orange jasmine with new shoots with (1) and without (2) an adhesive spray coating and those without new shoots with (3) and without (4) an adhesive spray coating. Treated plants were then placed in a screen cage (60 mesh, 40 × 50 × 60 cm) into which 20 ACP adult individuals (~3 mm in length) were placed. Observations were performed at 12, 24, and 36 h after ACP release. Assays were replicated three times. After the experiment, both the ACP and orange jasmine plants were transferred to another rearing net house.

#### 2.2.2. Field Experimentation Evaluation

Host plants were ~120 cm in height. The experiment was divided into four treatments (as for the indoor assay), and spraying was similarly repeated 2 or 3 times until the leaves were sticky to the touch. Three treated sections (1 m wide) were delineated along the hedge and interspersed with 0.5 m untreated gaps. Observations of trapped ACP were only performed after 12 and 24 h, as preliminary experiments showed that the adhesive applied to outdoor plants began to lose tackiness by 36 h, allowing occasional escape. ACP individuals trapped on 10 orange jasmine new shoots (that emerged after pruning) were counted for each replicate (30 shoots in total). Three replicate sections were also established as controls. For each control, one yellow sticky trap (20 × 24 cm, with yellow on both sides, purchased from Jiaduo Group (Hebi, Henan Province, China)) was installed at the midpoint of a 1 m wide unsprayed orange jasmine hedge section adjacent to sprayed plants. The trap was fixed vertically to a bamboo stick inserted into the ground. The trap was positioned according to methods described by Hall et al. [18], with slight modifications, such that its top edge was suspended ~20 cm above the canopy top, while the bottom edge was positioned close to, but not touching, the foliage.

### 2.3. Baseline ACP Sex Ratio

The study was performed within a net house planted with orange jasmine of ~120 cm height in Guangzhou, Guangdong province, China. A 60-mesh insect net was placed over the tops of the trees, within which ACP individuals were allowed to reproduce freely for more than three years. These orange jasmine plants were pruned once a month to provide tender shoots for ACP reproduction. ACP individuals were caught in glass tubes (diameter 2.5 cm) and sexed in the laboratory at 60× to determine the numbers of males and females. Sampling intervals were dynamically adjusted based on the visible ACP density. To ensure reliable gender ratio analysis, adult collections were initiated only when visual inspection confirmed ≥ 2 adult ACP individuals per actively growing orange jasmine shoot. Eight trials were performed. The sample size for each session was 60–150 insects to ensure statistical robustness. In total, 2522 ACP adults were sexed across all replications.

### 2.4. Assessment of ACP Directional Preference

**Figure 1:**
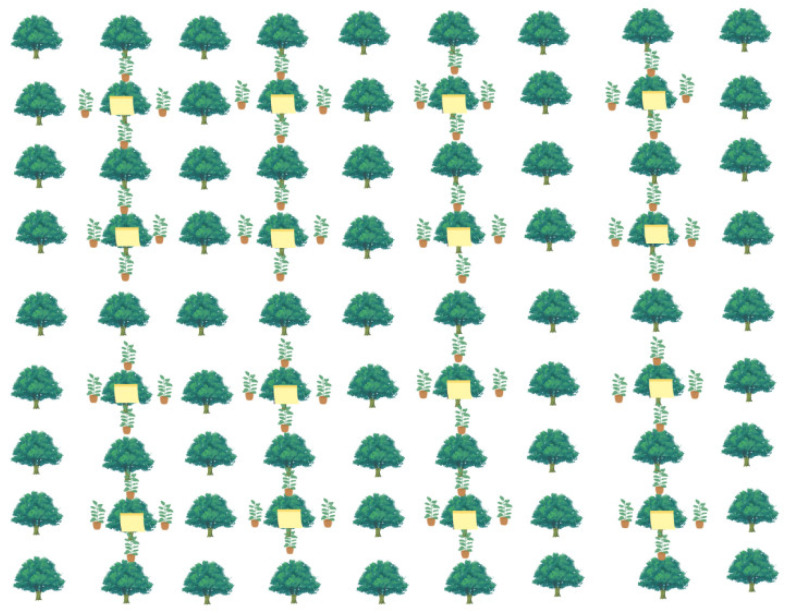
Schematic of potted *Murraya paniculata* plants and control groups in the orchard.

This component of the study was performed in an orchard in Xinhui, Guangdong Province, China, planted with *Citrus reticulata* “Chachi” trees of 2 y age, at a density of 4 × 5 m. Orange jasmines planted in pots with newly emerged adhesive-sprayed shoots were placed evenly in four directions beside the citrus trees. In each trial, orange jasmine traps were placed among four rows of trees, with four trees per row (Figure 1). Trees in each row were spaced at least two trees apart, and each row represented one replicate (four replicates with 16 trees). Replicates were spaced at least one row apart. One yellow sticky card (control) was placed on the trees in each direction. The number of ACP individuals on orange jasmine plants and sticky cards was counted after 24 h.

Pesticides (70% Acetamiprid 10,000 fold or 30% Thiamethoxam 1500 fold) were then sprayed on the orange jasmine to eliminate adult ACP individuals and their eggs. The orange jasmine was again pruned, fertilized, and watered. If orange jasmine plants grew poorly and sprouted no new shoots, they were replaced with new plants. Yellow sticky boards were replaced after each sampling. The experiment was performed five times (4 June, 19 June, 16 July, 15 August, and 6 September 2023) during the citrus tree non-shooting period.

### 2.5. Application in Monitoring ACP

Experiments were performed over 22 months in an orchard in Guangzhou, Guangdong Province, China that had been planted with *Citrus reticulata* “Shiyue Ju” trees of 4 y age at a density of 2 × 3 m. At the time of experimentation, the groves were experiencing an early stage of ACP infestation. Orange jasmine plants in pots with newly emerged adhesive-sprayed shoots were placed in rows alongside citrus trees. The numbers of ACP individuals on the orange jasmine and yellow sticky cards (controls) were counted after 24 h. Four replicates were performed. After sampling, pesticides (70% Acetamiprid 10,000 fold or 30% Thiamethoxam 1500 fold) were sprayed onto the orange jasmine to eliminate adult ACP individuals and their eggs.

Once the pesticide had evaporated, a dry adhesive was reapplied, and the yellow sticky traps were replaced with new ones. If orange jasmine plants grew poorly and sprouted no new shoots, they were replaced with new plants.

### 2.6. Data Analysis

One-way analysis of variance (ANOVA) was used to compare the means of two or more independent groups to identify significant differences between treatments. Means were compared by Tukey’s Student range test (equal variance) [19]. Differences were considered significant at *p* < 0.05. The SPSS v.17.0 software was used for analyses.

## 3. Results

### 3.1. Laboratory Experimentation

After 12 h, more than half of the ACP individuals had landed on the orange jasmine in all treatments; orange jasmine without sprayed new shoots had significantly fewer ACP individuals than orange jasmine with adhesive-sprayed new shoots. The numbers of ACP gradually increased over time. There was no significant difference in the numbers of ACP attracted to orange jasmine among treatments after 24 h. At 36 h, orange jasmine with unsprayed new shoots caught significantly more ACP individuals than other treatments. Additionally, the numbers of ACP on adhesive-sprayed orange jasmine with and without new shoots increased over time, peaking at 88.8%. Some ACP individuals remained on the screen and did not move or feed (Table 1).

### 3.2. Field Experimentation

Orange jasmine with young shoots attracted significantly more ACP than those without shoots, a trend consistent at both 12 h and 24 h. On these plants, the adhesive spray did not significantly affect capture numbers (e.g., at 24 h, 10.33 ± 0.88 individuals were caught with spray vs. 11.33 ± 0.67 without spray). In contrast, plants without shoots attracted significantly fewer ACP individuals (24 h: 5.00 ± 0.58 to 5.67 ± 0.67). The yellow sticky trap captured 6.24 ± 2.82 ACP adults. Significantly more female ACP individuals were attracted to orange jasmine with new shoots with and without the adhesive spray compared with orange jasmine without new shoots and yellow sticky traps (orange jasmine with new shoots with (72.41%) and without (71.86%) adhesive spray; orange jasmine with no new shoots with (51.27%) and without (53.79%) adhesive spray; yellow sticky traps (51.67%)). Additionally, after 24 h, more than half of the ACP individuals on adhesive-sprayed orange jasmine with and without new shoots treated were dead (57.0% and 54.28%); at this point, all ACP individuals on the yellow sticky traps had died. There was no significant difference in the numbers of ACP attracted to orange jasmine in treatments with and without the adhesive spray (Table 2).

### 3.3. ACP Sex Ratio

Over the course of the survey, a total of 2522 ACP adults were collected, comprising 820 females and 1702 males. The sex ratio (percentage of males) varied between sampling dates, from 62.78% to 74.62%. ACP was male-biased among the captured populations (Table 3).

### 3.4. ACP Directional Preference

The directional preferences of ACP when feeding and moving were surveyed during the cooler morning/afternoon periods on 4 June and 19 June, 16 July, 15 August, and 6 September 2023 (Table 4). The highest number of ACP individuals, consistently recorded in the south (4 June: 13.00 ± 0.41; 19 June: 9.75 ± 0.85; 16 July: 9.50 ± 0.65), was significantly greater than in the west and north on 4 June (west: 6.75 ± 0.75; north: 9.50 ± 0.65) and exceeded that in all other directions on 19 June (east: 7.00 ± 0.91; west: 6.50 ± 0.65; north: 6.00 ± 0.41). Although the overall activity declined seasonally, the activity in the south was greater than in the north in August (5.75 ± 0.63 vs. 3.00 ± 0.41) and September (5.75 ± 0.48 vs. 2.75 ± 0.48). Aggregated data revealed numerically greater activity in the south (34.00 ± 5.39), but there were no statistically significant (*p* > 0.05) directional preferences across dates (east: 26.00 ± 5.10; west: 22.20 ± 2.35; north: 20.20 ± 4.99).

### 3.5. ACP Detection and Monitoring

**Figure 2:**
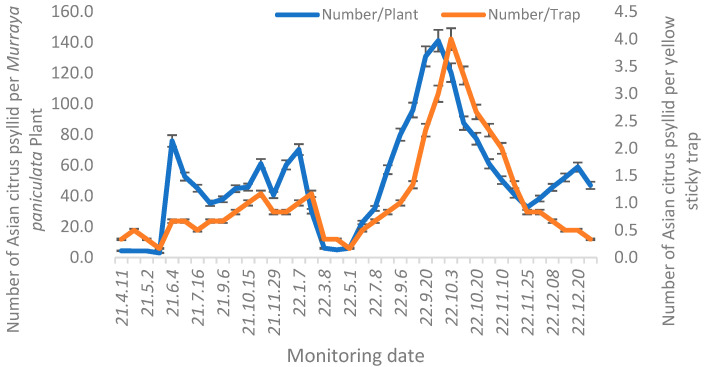
Dynamic detection of Asian citrus psyllid using *Murraya paniculata* adhesive and yellow sticky traps.

ACP populations peaked in June and November 2021 and January and October 2022. Despite two distinct population surges in ACP, the cumulative abundance in 2021 was lower than that recorded in 2022. In 2022, a small peak appeared earlier than in 2021, and the numbers increased significantly from May to October (Figure 2). The numbers of adult ACP attracted to a single yellow sticky trap were significantly lower than those attracted to an individual orange jasmine plant with new shoots. The trends in ACP population dynamics detected by both methods were generally similar, deviating from February to March and late November to December of 2022. During this period, orange jasmine with new shoots recorded a small peak in ACP populations, while the numbers on yellow sticky traps gradually declined.

## 4. Discussion

In agricultural pest management, trap cropping is a cost-effective way to reduce crop damage and decrease the need for traditional pesticides [20,21]. Trap crops attract insects for feeding and oviposition, but they might also function as reservoirs of pathogens that potential vectors might carry [22].

We demonstrate that orange jasmine with adhesive-sprayed new shoots was effective in trapping ACP adults, particularly females. Several studies have investigated trap cropping strategies for ACP management. Patt and Sétamou [17] and Hall et al. [23] studied the attraction of ACP to orange jasmine in indoor conditions. de Carvalho et al. [24] top-pruned sweet orange trees around orchards to boost new shoot growth and investigated their concentrated trapping effects on ACP and their role in the prevention and control of HLB. However, their approach was complicated by their inability to prune all fruit trees year-round, and the frequent topping of orange trees risks reducing the yield, limiting the success of this approach [24].

Adhesives are widely applied in plant protection as colored sticky cards and traps [25,26]. We used orange jasmine with new shoots to attract and trap ACP; the application of a sticky coating (adhesive) also avoided the escape of ACP mentioned by Patt and Sétamou [17] and Hall et al. [23]. Once an adult ACP was trapped, it was unable to feed and struggled to break free. The psyllid’s death in this work was likely initiated by exhaustion and hunger and was ultimately ensured by a timely pesticide spray that killed all trapped psyllids.

A primary risk of using orange jasmine as a trap crop lies in its potential to support the reproduction of *C*Las-positive ACP. Although often asymptomatic, orange jasmine can serve as a pathogen reservoir [27]. ACP individuals acquiring the pathogen through feeding on infected plants may subsequently transmit the disease, and their offspring developing on such plants could also become infected [28], potentially leading to the massive proliferation of infective ACP. Therefore, the most critical aspect in practical applications is to ensure timely pesticide spraying to eliminate ACP before the adhesive loses its viscosity. This must be coupled with the use of *C*Las-free nursery stock and regular plant replacement to ensure that orange jasmine acts as a lethal ecological sink, preventing it from becoming a source of HLB transmission.

Trapping was more successful in the laboratory than it was in the field, possibly because of the uneven adhesive distribution on plants caused by the large hedge area, environmental factors (e.g., direct sunlight, wind, and dust), and reduced spray adhesiveness after 24 h of application (in the preliminary experiment, observations at the 36 h mark determined adhesive properties to be suboptimal). For field application, frequent adhesive resprays were needed to maintain the efficacy of the trap crop.

An accurate assessment of sex ratios is important for biological control initiatives. Several studies have reported ACP sex ratios. Sule et al. [29] reported a female-to-male ratio of 1:0.65, while Aubert and Quilici [30] reported a near-equal ratio. We report a male-biased ratio, with an average female-to-male ratio of 1:2.08, similar to Mann et al. [31].

A persistent sex bias in ACP populations creates a vulnerability whereby population growth depends on female recruitment. Our finding that orange jasmine shoots selectively attract females provides a mechanism by which this weakness can be exploited. Removing a single gravid female prevents hundreds of offspring and exacerbates mate-finding challenges in male-dominated populations. Such female-focused tactics—unlike broad insecticides—leverage the intrinsic population structure for sustainable control, potentially disrupting HLB transmission at its source.

Management strategies for ACP populations often depend on effective monitoring systems, and yellow sticky traps have been widely used [32,33]. However, a key limitation of these traps is their ineffectiveness in detecting sparse adult populations [18,34]. ACP individuals need new shoots to reproduce. After mating, they then begin searching for suitable oviposition sites [35,36]. Citrus plants grow new shoots four or five times annually [11], and growers typically leave spring and autumn shoots and remove summer and winter shoots. The time available for ACP individuals to lay their eggs is relatively short within the orchard. If new shoots on (sacrificial) trap plants could be provided during unsuitable periods, many mature, female ACP individuals are likely to be attracted to them to lay eggs [33,35,36,37] and be killed. This feature is responsible for our traps’ effectiveness in capturing ACP at low densities, enabling the high-accuracy and high-reliability monitoring of their population dynamics.

Because we did not directly assess actual ACP populations on citrus trees, the absolute number of trapped ACP individuals may not correspond on a 1:1 basis to the absolute population present on trees at any specific time. Nevertheless, the trends and dynamics of trapped populations—such as peaks and troughs in activity—serve as a robust and reliable indicator of the relative activity levels and presence of ACP within the entire orchard system, including on citrus trees.

The direction in which ACP migrates is intrinsically linked to trends in the space and time of HLB occurrence. The height of citrus trees and the small size of ACP made it difficult to perform quantitative surveys. We used citrus tree non-flushing periods and introduced orange jasmine with new shoots as a food and oviposition source to determine ACP’s migration direction preferences. We demonstrate that ACP exhibits significant phototropic behavior, preferring to feed and rest on the south- and east-facing surfaces of trees. These results are similar to those of Sétamou et al. [38], who reported significantly more immature ACP individuals in the southeastern quadrant of trees than in other parts of the canopy. This suggests that, during dispersal or migration, the risk of ACP spreading HLB disease is greater in the southeastern direction within an orchard. This might also explain the distribution of ACP hotspots surrounding Los Angeles (a rapid and strongly asymmetrical spread to the south and east) and the non-random spatial and temporal distribution in Southern California [39]. The long-distance dispersal of ACP might also be influenced by its directional preferences and factors such as wind [40].

In our 800-acre citrus plantation base, an ~20-acre area was divided into eastern and western units by a north–south service road, with the southern boundary forming a natural barrier through an east–west-trending gully. Monitoring data from the fourth year of planting revealed a significant infection gradient (unpublished): the HLB infection rate in the southeastern aspect reached ~40%, while the southwestern aspect showed an infection rate of ~20%. In contrast, the northwestern and northeastern aspects exhibited significantly lower incidence rates, both below 10%. This spatial distribution pattern suggests that the particularly severe infection in southeastern areas may be linked to the directional foraging and habitat preferences of ACP, possibly influenced by the specific wavelength and incidence angle characteristics of morning sunlight, which appear to enhance ACP attraction and facilitate pathogen transmission more effectively than afternoon light conditions.

Future research should explore the efficacy of non-host or suboptimal host plants that are highly attractive for oviposition but unable to support complete development and colonization, such as *Bergera koenigii* (curry leaf) [41], in combination with adhesive trapping. This could further enhance the safety and sustainability of the trap cropping strategy. However, the effectiveness of any single trapping strategy against ACP may be limited, particularly given the highly efficient transmission mechanism of citrus HLB. Therefore, future studies incorporating research on light—including wavelength, polarity, and intensity—may offer promising novel avenues for improvements in ACP control efficacy.

## 5. Conclusions

We report the results of a trap cropping method based on the adhesive spraying of new shoots of orange jasmine to attract ACP. This approach also facilitates ACP population monitoring and assessments of directional movement, and it appears to be more effective than yellow sticky traps. Trap cropping represents a practical tool for controlling ACP in citrus groves (managed or otherwise). It is particularly effective in urban areas and unmanaged citrus refugia, which serve as primary sources of ACP infestations for commercial groves. However, determining the optimal deployment timing and efficacy of this method for controlling HLB in the field requires further investigation.

## Figures and Tables

**Table 1:** The trapping effects of spraying adhesive on *Murraya paniculate* against *Diaphorina citri*.

	Trap Number on *Murraya paniculate*			Mortality Rate		
	12 h	24 h	36 h	12 h	24 h	36 h
Shoot with spray	13.33 ± 0.88 a	16.33 ± 0.58 a	18.00 ± 0.58 ab	51.8%	75.9%	88.8%
Shoot without spray	13.67 ± 0.33 a	15.33 ± 0.33 a	18.33 ± 0.33 a	0%	0%	0%
No shoot with spray	11.00 ± 0.58 b	15.33 ± 0.88 a	16.67 ± 0.33 bc	49.07%	63.41%	77.31%
No shoot without spray	12.33 ± 0.33 ab	16.00 ± 1.00 a	16.00 ± 0.58 c	0%	0%	0%

Survival number refers to the total number of *Diaphorina citri* that were on the *Murraya paniculate* plant and alive, excluding the *Diaphorina citri* that stayed on the cage all day long (±SE). Mortality rate = (N_initial − N_alive)/N_initial, where N_initial is the total number of *Diaphorina citri* on the *Murraya paniculata* plant at the start, and N_alive is the number of insects surviving. Different letters within a column indicate significant differences (*p* < 0.05).

**Table 2:** The effects of spraying adhesive on the green belt of *Murraya paniculate* against *Diaphorina citri*.

	12 h Trap Number/10 Young Shoots	12 h Mortality Rate	24 h Trap Number/ 10 Young Shoots	24 h Female Ratio	24 h Mortality Rate
Shoot with spray	7.66 ± 0.88 a	13.04%	10.33 ± 0.88 a	72.41%	57.0%
Shoot without spray	8.33 ± 0.88 a	0.00%	11.33 ± 0.67 a	71.86%	0.00%
No shoot with spray	2.33 ± 0.33 b	14.29%	5.00 ± 0.58 b	51.27%	54.28%
No shoot without spray	3.00 ± 0.58 b	0.00%	5.67 ± 0.67 b	53.79%	0.00%
Yellow sticky trap control	2.67 ± 0.67 b	100%	6.24 ± 2.82 b	51.67%	100%

Mortality rate = number of *Diaphorina citri* dead/number of *Diaphorina citri* captured. To avoid disturbing the leaves of the *Murraya paniculate* plants, the sex ratio of the captured *Diaphorina citri* was calculated uniformly only at 24 h. Different letters within a column indicate significant differences (*p* < 0.05).

**Table 3:** Investigation of the sex ratio of Asian citrus psyllid by *Murraya Paniculata* adhesive.

Survey Date	Female	Male	Sex Ratio (% Male)
13 July 2023	150	290	65.91
20 July 2023	120	210	63.63
25 July 2023	86	195	69.40
26 July 2023	67	197	74.62
27 July 2023	80	201	71.53
6 August 2023	120	266	68.91
9 August 2023	130	230	63.89
10 August 2023	67	113	62.78
Total	820	1702	67.49

**Table 4:** The directional preferences of citrus psyllids detected by *Murraya Paniculata* adhesive.

Survey Date	East	West	South	North
4 June 2023	11.25 ± 1.11 ab	6.75 ± 0.75 c	13.00 ± 0.41 a	9.50 ± 0.65 b
19 June 2023	7.00 ± 0.91 b	6.50 ± 0.65 b	9.75 ± 0.85 a	6.00 ± 0.41 b
16 July 2023	5.75 ± 0.85 bc	6.25 ± 1.11 bc	9.50 ± 0.65 a	4.50 ± 0.64 c
15 August 2023	4.75 ± 0.85 ab	4.50 ± 0.65 ab	5.75 ± 0.63 a	3.00 ± 0.41 b
6 September 2023	4.25 ± 0.75 ab	4.00 ± 0.71 ab	5.75 ± 0.48 a	2.75 ± 0.48 b
Total	26.00 ± 5.10 a	22.20 ± 2.35 a	34.00 ± 5.39 a	20.20 ± 4.99 a

Different letters within a row indicate significant differences (*p* < 0.05).

## Data Availability

The original contributions presented in this study are included in the article. Further inquiries can be directed to the corresponding author.
